# Curcumin-incorporated EGCG-based nano-antioxidants alleviate colon and kidney inflammation via antioxidant and anti-inflammatory therapy

**DOI:** 10.1093/rb/rbae122

**Published:** 2024-10-17

**Authors:** Qingqing Pan, Li Xie, Huang Zhu, Zhihui Zong, Di Wu, Rong Liu, Bin He, Yuji Pu

**Affiliations:** School of Preclinical Medicine, Chengdu University, Chengdu 610106, China; School of Preclinical Medicine, Chengdu University, Chengdu 610106, China; School of Preclinical Medicine, Chengdu University, Chengdu 610106, China; Department of Pharmaceutical Engineering, Bengbu Medical University, Bengbu 233030, China; Meat Processing Key Laboratory of Sichuan Province, School of Food and Biological Engineering, Chengdu University, Chengdu 610106, China; School of Preclinical Medicine, Chengdu University, Chengdu 610106, China; National Engineering Research Center for Biomaterials, College of Biomedical Engineering, Med-X Center for Materials, Sichuan University, Chengdu 610064, China; National Engineering Research Center for Biomaterials, College of Biomedical Engineering, Med-X Center for Materials, Sichuan University, Chengdu 610064, China

**Keywords:** anti-oxidation, anti-inflammation, curcumin, bioavailability, epigallocatechin gallate

## Abstract

Natural remedies are gaining attention as promising approaches to alleviating inflammation, yet their full potential is often limited by challenges such as poor bioavailability and suboptimal therapeutic effects. To overcome these limitations, we have developed a novel nano-antioxidant (EK) based on epigallocatechin gallate (EGCG) aimed at enhancing the oral and systemic bioavailability, as well as the anti-inflammatory efficacy, of curcumin (Cur) in conditions such as acute colon and kidney inflammation. EK is synthesized using a straightforward Mannich reaction between EGCG and L-lysine (K), resulting in the formation of EGCG oligomers. These oligomers spontaneously self-assemble into nanoparticles with a spherical morphology and an average diameter of approximately 160 nm. *In vitro* studies reveal that EK nanoparticles exhibit remarkable radical-scavenging capabilities and effectively regulate redox processes within macrophages, a key component in the body’s inflammatory response. By efficiently encapsulating curcumin within these EK nanoparticles, we create Cur@EK, a formulation that demonstrates a synergistic anti-inflammatory effect. Specifically, Cur@EK significantly reduces the levels of pro-inflammatory cytokines TNF-α and IL-6 while increasing the anti-inflammatory cytokine IL-10 in lipopolysaccharide-stimulated macrophages, highlighting its potent anti-inflammatory properties. When administered either orally or intravenously, Cur@EK shows superior bioavailability compared to free curcumin and exhibits pronounced anti-inflammatory effects in mouse models of ulcerative colitis and acute kidney injury. These findings suggest that the EK nano-antioxidant platform not only enhances the bioavailability of curcumin but also amplifies its therapeutic impact, offering a promising new avenue for the treatment and management of inflammation in both oral and systemic contexts.

## Graphical abstract

**Figure:**
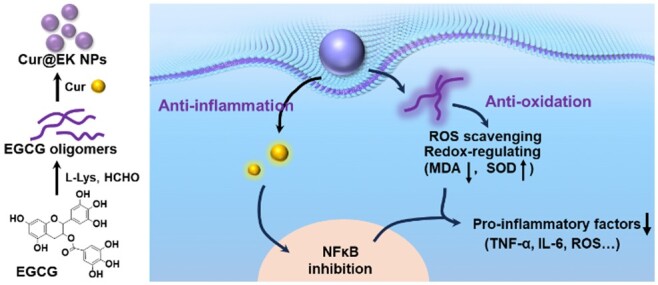

## Introduction

Inflammation is the body’s natural response to injury, infection, or harmful stimuli [1, 2]. It is a complex process involving the immune system, which aims to protect the body by removing harmful agents, such as pathogens, damaged cells, and irritants, and initiating the healing process. Inflammation can be acute, lasting a few days, or chronic, persisting for weeks, months, or even years. Traditional treatments of inflammation include non-steroidal anti-inflammatory drugs and corticosteroids. These medications for inflammation, while effective, can have several disadvantages [3, 4]. These include potential side effects, long-term health risks, and interactions with other medications. For example, corticosteroids can lead to weight gain and increased susceptibility to infections because they can affect glucose metabolism and fat distribution and suppress the immune system. Therefore, novel therapeutics with better biosafety profile to manage inflammation are highly needed.

Natural remedies, including herbal supplements and omega-3 fatty acids, are emerging as promising alternative approaches to alleviating inflammation [5–9]. Curcumin (Cur) is a natural compound widely utilized in Chinese medicine due to its impressive anti-inflammatory properties, antioxidant activity and potential for cancer prevention [10–14]. However, its biomedical application *in vivo* is significantly hindered by its poor bioavailability. Firstly, Cur is inadequately absorbed in the gastrointestinal tract owing to its hydrophobic nature, which restricts its dissolution and uptake into the bloodstream. Secondly, once absorbed, Cur undergoes rapid metabolism by the liver and intestinal wall, resulting in the production of metabolites with potentially reduced bioactivity. Thirdly, Cur is swiftly eliminated from the body through feces and urine, reducing its duration of systemic circulation. Therefore, designing drug delivery systems to enhance the bioavailability and anti-inflammatory effects of Cur is appealing, as it could further promote its exploration and clinical application in treating inflammation.

Recently, epigallocatechin gallate (EGCG)-based nanoplatforms have demonstrated significant potential in drug delivery and antioxidation [15–18]. Notably, the intrinsic antioxidant and anti-inflammatory properties of EGCG could enhance the therapeutic efficacy of payloads across various biomedical applications [19, 20]. The phenol groups in EGCG facilitate strong coordination interactions with transition metal ions, allowing the formation of coordination nanoparticles [21–23]. However, these nanosystems are sensitive to interference from biological molecules such as glutathione (GSH) and amino acids, which can also bind to these metal ions. Additionally, the aromatic nature of EGCG enables efficient Cur loading due to π–π stacking interactions between EGCG and Cur [24, 25]. Consequently, employing a polymer-based EGCG nanosystem could offer promising results in curcumin delivery and enhanced inflammation alleviation, thanks to its superior stability.

**Scheme 1.**
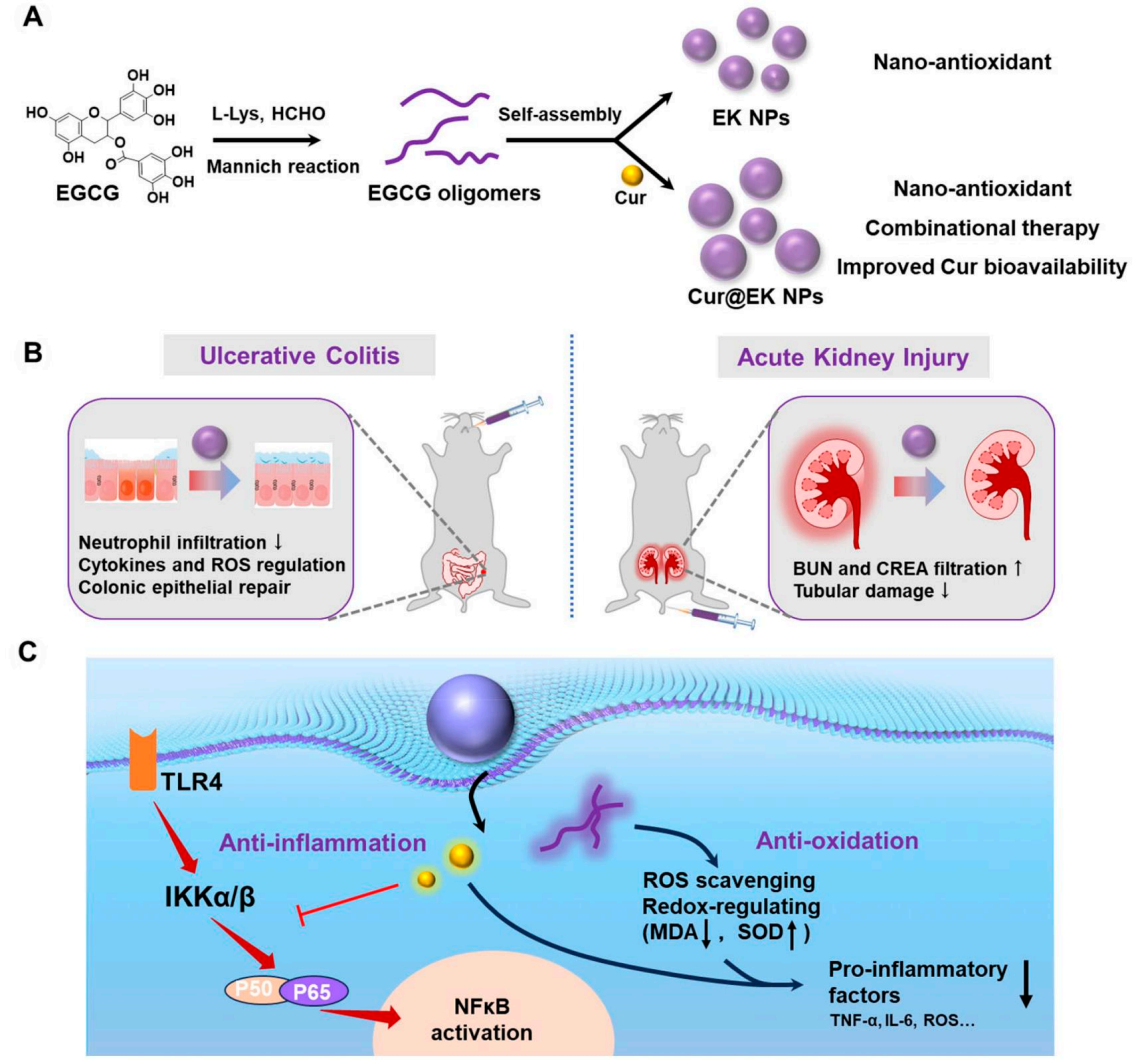
Illustration of Cur-loaded EK nanoparticles for enhanced anti-inflammation therapy of acute colitis and kidney injury. (**A**) The preparative scheme of blank and Cur-loaded EK NPs. (**B**) The oral and intravenous administration of Cur@EK for ulcerative colitis and AKI therapy. (**C**) The anti-oxidant and anti-inflammatory mechanisms of Cur@EK in activated macrophages at sites of inflammation.

In this study, we developed an EGCG-based nanoplatform to enhance the oral and systemic bioavailability and therapeutic efficacy of Cur for treating ulcerative colitis and acute kidney injury (Scheme 1). A series of EK nanoparticles (NPs) were synthesized through Mannich reactions between EGCG and L-lysine (K). The resulting EGCG oligomers self-assembled into EK NPs, which exhibited significant *in vitro* antioxidant and anti-inflammatory effects by scavenging reactive oxygen species (ROS) and regulating redox-associated molecules in macrophages. Cur-loaded EK nanoparticles (Cur@EK) demonstrated controlled drug release and a synergistic anti-inflammatory effect in activated macrophages. The *in vivo* efficacy of EK and Cur@EK in mitigating inflammation was evaluated in a dextran sulfate sodium (DSS)-induced colitis model and a glycerol-induced acute kidney injury (AKI) model. These findings suggest that EK NPs represent a promising nanoplatform for enhancing the therapeutic efficacy of antioxidant and anti-inflammatory polyphenols in treating inflammation.

## Experimental 

### Materials and methods

Epigallocatechin gallate (EGCG, 98%) and L-lysine (Lys, 98%) were purchased from Aladdin. Curcumin (Cur, 98%) was obtained from Sigma-Aldrich. H_2_O_2_ (30 wt.%), lipopolysaccharide, 2,2′-azino-bis(3-ethylbenzothiazoline-6-sulfonic acid) ammonium salt (ABTS, 98%) and 1,1-diphenyl-2-picrylhydrazyl (DPPH, 98.5%) were from Macklin. Dextran sulfate sodium salt (DSS, 40 kDa) was purchased from Heowns (Tianjin, China). Formaldehyde (37 wt.% in water) and other organic solvents were purchased from Chron (Chengdu, China). The RAW264.7 macrophage cells were obtained from the National Collection of Authenticated Cell Cultures.

### Preparation of EK nanoparticles

The preparation of EK NPs was performed according to a previous study with minor modification [26]. The detailed feeding amounts for preparing EK nanoparticles were summarized in Supplementary Table S1. Briefly, formaldehyde was added into EGCG solution in water. L-lysine solution was then dropwise added into the solution. The blend underwent stirring for 2 h, followed by the retrieval of EK nanoparticles via centrifugation. Subsequently, they were washed thrice with water prior to being stored in the refrigerator. The characterization methods are consistent with those used in our recent study [20].

### *In vitro* antioxidant study

EK and DPPH (or ABTS) were mixed and allowed to incubate for 30 min in the dark. Afterward, the absorbance was measured using a microplate reader at 517 nm for DPPH and 734 nm for ABTS. The antioxidant activity was assessed based on the absorbance readings according to a prior investigation [27].

### Curcumin loading

EK (20 mg) was dissolved in 1 ml DMSO and curcumin (4.0 mg) in 0.5 ml dimethyl sulfoxide (DMSO). The two solutions were mixed and slowly added into 10 ml of distilled water while stirring. After stirring for 2 h, the mixture was dialyzed against deionized water (molecular weight cutoff, MWCO 2000 Da) for 2 days. Cur-incorporated NPs were obtained after freeze drying. The drug loading contents and encapsulation efficiencies were calculated using fluorescence spectroscopy. Briefly, the curcumin-loaded nanoparticles were weighed, re-dissolved in DMSO, and their fluorescence intensity was measured to assess the curcumin loading capacity.

$$\mathrm{Curcumin}\;\mathrm{loading}\;\mathrm{capacity}\;(\%)\;=\;\frac{\text{Weight}\;\text{of}\;\text{curcumin}\;\text{in}\;\text{NPs}}{\text{Weight}\;\text{of}\;\text{curcumin-loaded}\;\text{NPs}}\;\times \;100\%$$

### *In vitro* drug release

The *in vitro* release of Cur from Cur@EK at 37 °C was evaluated using a dialysis method. To simulate gastrointestinal pH conditions, synthetic gastric, intestinal, and colonic fluids were utilized, with pH values of 1.2, 6.8 and 7.4, respectively. The release periods were set to 2 and 4 h for the gastric and intestinal fluids, while the colonic fluid was used for a 42-hour release period. At predetermined time points, 1 ml of the release medium was removed and replaced with an equal volume of fresh medium. The amount of Cur released was quantified by fluorescence spectroscopy (F-700, Hitachi, Japan).

To mimic drug release in blood, the experiment was conducted at pH 7.4, following the same protocol.

### Cell viability and intracellular ROS level

Macrophages were cultured with medium containing 300 μM H_2_O_2_ for 12 h and then co-incubated with various NPs (100 μg/ml) for an additional 12 h. The cell viability was measured using a typical MTT assay [28]. For measuring the intracellular ROS level, cells were first treated with H_2_O_2_ for 12 h and then exposed to NPs for 12 h, followed by treatment with 2′,7′-dichlorodihydrofluorescein diacetate (DCFH-DA) for 20 min [29]. The intracellular fluorescence intensity was measured by flow cytometry and confocal laser scanning microscopy.

### *In vitro* anti-inflammatory effect in macrophages

Macrophages were treated with lipopolysaccharide (LPS, 1 μg/ml) for 4 h and then co-cultured with curcumin-loaded NPs for 20 h. The supernatant was collected to determine the levels of tumor necrosis factor-α (TNF-α), interleukin-6 (IL-6) and IL-10 using enzyme-linked immunosorbent assay (ELISA) kits (Catalog numbers PT512, PI326, PI522, respectively; all from Beyotime Biotechnology Inc).

### *In vivo* anti-colitis therapy

Balb/c female mice (body weight: about 20 g) were acclimated for one week and then treated with 3.0% DSS (w/v) to establish mouse model with ulcerative colitis [30]. Animal experiments were approved by Sichuan University’s Animal Care and Use Committee (No. KS2022889). On day 4, colitis mice were randomly divided into four groups and gavaged with saline, curcumin, EK and Cur@EK (curcumin: 50 mg/kg). EK and Cur@EK were suspended in saline. Curcumin was first dissolved in a small volume of DMSO and castor oil, followed by dilution with saline in a ratio of 5% DMSO, 5% castor oil and 90% saline (v/v/v). Mice treated with DSS-free drinking water were used as a control group. During the therapy period, the disease activity index (DAI) and changes in body weight of the mice were recorded daily [31]. On day 11, the mice were euthanized under anesthesia. Colons and other major organs were collected, and colon lengths were measured. Organ samples were then stained with H&E to evaluate therapeutic effectiveness and organ toxicity.

### Immune and epithelial repair evaluation in vivo

Immunochemical analysis was used to evaluate myeloperoxidase (MPO) expression and the levels of ZO-1 and Occludin-1 in inflamed colons. The concentrations of cytokines including TNF-α, IL-6, and IL-10 in the colon were measured using ELISA [32]. Colon tissues were stained with periodic acid-Schiff (PAS) kits to evaluate the epithelial repair.

### *In vivo* acute kidney injury therapy

Balb/c mice underwent a 15-hour period of water deprivation followed by the induction of acute kidney injury (AKI) by injecting 50% glycerol (8 ml/kg) into their hind limbs [33]. Animal experiments were approved by Sichuan University’s Animal Care and Use Committee (No. KS2023625). At 2 h post glycerol injection, mice with AKI were randomly assigned to four groups and intravenously injected with saline, curcumin, EK and Cur@EK (Cur: 10 mg/kg). EK and Cur@EK were suspended in saline. Curcumin was first dissolved in a small volume of DMSO and castor oil, followed by dilution with saline in a ratio of 2% DMSO, 8% castor oil, and 90% saline (v/v/v). Healthy mice were used as a control group. At 24 h post glycerol administration, blood samples were collected to measure blood urea nitrogen (BUN) and creatinine (CRE) levels. Additionally, kidney and other major organ tissues were subjected to H&E staining to evaluate tissue damage.

### Statistical analysis

Statistical analysis was performed utilizing a Student’s *t*-test, and the difference was denoted at * *P *<* *0.05, ** *P *<* *0.01 and *** *P *<* *0.001.

## Results and discussion

### Preparation and characterization of EK NPs

**Figure 1.**
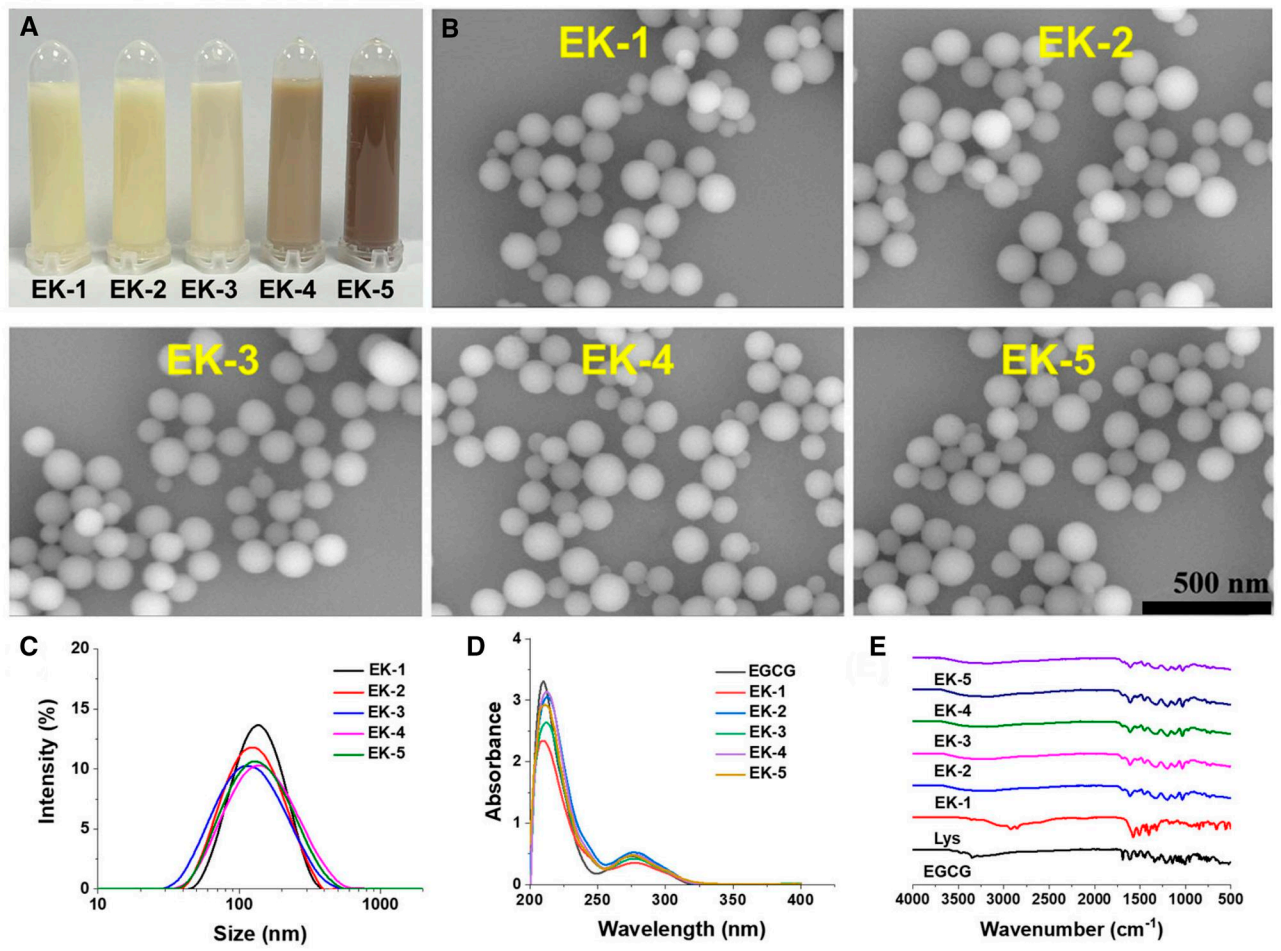
Preparation and characterization of EK NPs. (**A**) Photographs of freshly prepared EK nanoparticle suspensions. (**B**) SEM images of EK NPs prepared with various feeding molar ratios of EGCG/lysine. (**C**) DLS size distributions of EK NPs. (**D**) UV-vis spectra of EK NPs. (**E**) FT-IR spectra of different NPs.

The EK nanoparticles were easily synthesized using a conventional Mannich reaction in an aqueous solution involving EGCG, L-lysine, and formaldehyde [26]. L-lysine solution was added to the solution of EGCG and formaldehyde. A range of EK NPs were produced by keeping the concentrations of EGCG and formaldehyde constant while varying the amount of L-lysine (Supplementary Table S1). Within 2 h, colloidal nanoparticle suspension was observed (Figure 1A). The darker colors for EK-4 and EK-5 were possibly due to the partial oxidation of phenol groups at weak alkaline condition [34, 35], where the pH values at the end point were 8.02 and 8.65, respectively (Supplementary Table S1). Scanning electron microscopy (SEM) images clearly revealed that EK NPs shared the spherical morphology and showed a comparable average size of 120 nm with narrow distribution (Figure 1B). The hydrodynamic diameters of EK NPs were measured by dynamic light scattering (DLS) technology to be around 160 nm (Figure 1C) and the zeta potentials were about −22 mV (Supplementary Figure S1). The formation of oligomeric EGCG was verified using MALDI-TOF MS (Supplementary Figure S2). The results manifested that the molecular weights of EGCG oligomers were high as 2052 Da. The molecular weight increase of 471 is attributed to the addition of the CH_2_EGCG moiety via Mannich reaction, in which CH_2_ is from formaldehyde [26]. Both EGCG and EK NPs shared similar UV-vis absorption peaks, particularly strong at around 280 nm (Figure 1D), which are the typical absorption of benzene moieties in EGCG [36]. In the FT-IR spectrum of EGCG, the characteristic peak at 1690 cm^−1^ was attributed to carbonyl stretching (Figure 1E) [37]. EK NPs exhibited diminished absorption at this wavenumber, indicating the consumption of carbonyl groups in the reactions. In addition, the minimal size fluctuation of EK in PBS and serum indicates its strong physiological stability (Supplementary Figure S3).

### *In vitro* ROS scavenging and cytocompatibility

**Figure 2.**
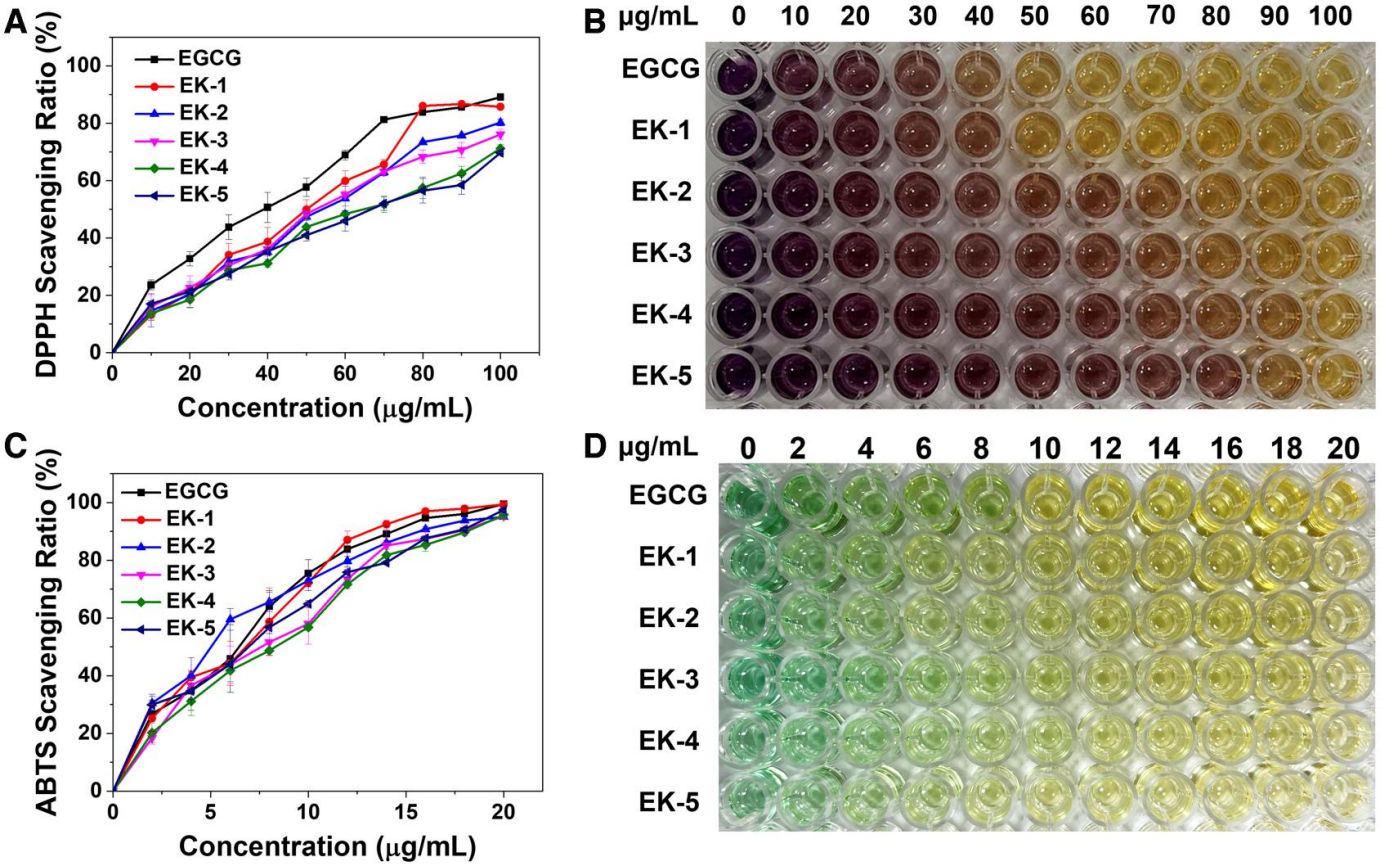
Anti-oxidant capacity of EGCG and EK NPs. (**A, B**) DPPH scavenging ratios and images of EK NPs. (**C, D**) ABTS scavenging ability and images of EK NPs (*n *=* *3).

The antioxidant capacity of EK NPs was assessed using 1,1-diphenyl-2-picrylhydrazyl (DPPH) and 2,2′-azino-bis(3-ethylbenzothiazoline-6-sulfonic acid) ammonium salt (ABTS) assays [38]. EGCG was used as a reference antioxidant. The DPPH assay measures radical scavenging by detecting UV-vis absorption at 517 nm. Figure 2A and B depicted that EK NPs and EGCG exhibited concentration-dependent radical scavenging, with EK-1 and EK-2 showing higher scavenging percentages at a concentration of 100 μg/ml due to a higher content of EGCG. However, at lower concentrations, EK NPs showed lower DPPH scavenging compared to EGCG, likely due to depletion of phenol groups during nanoparticle preparation. Furthermore, EK NPs exhibited slightly weaker ABTS radical scavenging compared to EGCG (Figure 2C and D), with EK-1 and EK-2 again demonstrating stronger scavenging tendencies.

The cytocompatibility of EK NPs was then studied. L929 and RAW264.7 cells were co-cultured with EK NPs for 24 h, followed by assessment of cell viability using an MTT assay. As shown in Supplementary Figures S4 and S5, EK NPs exhibited good cytocompatibility at the tested concentrations. L929 and RAW264.7 cells showed a viability of more than 80% upon co-incubation with 200 μg/ml EK. Similarly, more than 95% colonic epithelial cells were alive after EK-5 treatment (Supplementary Figure S6). These results suggested EK not only maintained the *in vitro* antioxidant capability but also possessed great cytocompatibility.

### ROS scavenging and redox-regulation in the cellular level

**Figure 3.**
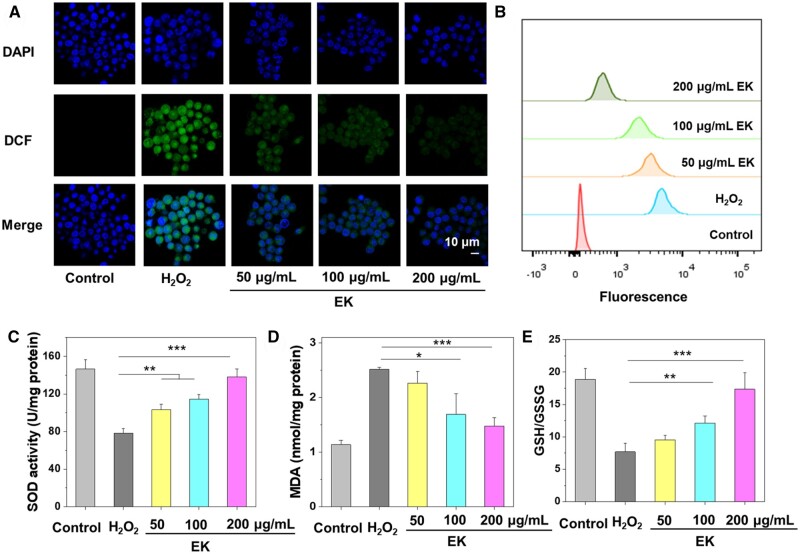
Confocal laser scanning microscopy (CLSM) images (**A**) and flow cytometry analysis (**B**) of H_2_O_2_ and EK treated RAW264.7 cells; DCFH-DA was used as a fluorescence indicator of intracellular ROS. (**C–E**) Levels of SOD, MDA and GSH/GSSG ratios in macrophages treated with H_2_O_2_ and EK NPs (*n *=* *5. *P <* *0.05; **P <* *0.01; ***P <* *0.001).

Motivated by the encouraging *in vitro* antioxidant capabilities observed in EK NPs, we tested the capability to scavenge intracellular ROS, a notorious factor in inflammation [39, 40]. Macrophages were exposed to exogeneous H_2_O_2_ to upregulate the intracellular ROS levels since H_2_O_2_ is a water-miscible molecule and can freely diffuse into cells. A fluorescent probe DCFH-DA was used to probe the intracellular ROS level, and strong green fluorescence in the cells indicates elevated intracellular level of ROS. As shown in Figure 3A, H_2_O_2_ treatment remarkably increased ROS levels in macrophages and EK-5 (denoted as EK for clarity in the following study) reversed this trend in a concentration-dependent manner. This ROS scavenging effect of EK NPs was further confirmed by flow cytometry analysis (Figure 3B). The average fluorescence intensity of macrophages treated with 200 μg/ml EK decreased 10 times compared to the H_2_O_2_ group, suggesting a potent efficacy in ROS downregulation in macrophages. Furthermore, the upregulated ROS level is associated with intracellular oxidative stress, which could induce cell death [41, 42]. The capability of EK NPs to reverse the cell death induced by exogeneous H_2_O_2_ was further studied. H_2_O_2_ treatment led to a low viability of 68.8% due to the intracellular oxidative stress (Supplementary Figure S7). EK NPs treatment led to increased cell viability and EK-5 exhibited a high viability of 82.3%, higher than that of EGCG group (73.3%). EGCG displayed better oxidative stress alleviation and cell survival than EGCG due to the increased cytotoxicity of free EGCG at an elevated level.

To further understand the anti-oxidant effects of EK, we measured the levels of the intracellular antioxidant enzyme superoxide dismutase (SOD) [43]. EK treatment demonstrated a concentration-dependent upregulation of SOD levels compared with cells treated with H_2_O_2_ (Figure 3C). Specifically, 200 μg/ml EK treatment led to an increase of SOD activity to 138.1 U/mg protein, nearly two times of H_2_O_2_ group (78.2 U/mg protein) and close to the level of control group (146.3 U/mg protein). In addition, malondialdehyde (MDA) is a biomarker to assess the level of lipid peroxidation in cells [44]. Elevated level of MDA indicates increased oxidative stress and lipid damage, which can be associated with pathological conditions such as inflammation, cardiovascular diseases and neurodegenerative disorders. As shown in Figure 3D, H_2_O_2_ stimulation led to the upregulation of the MDA level in macrophages, which was effectively reduced by EK treatment at a higher concentration (100 and 200 μg/ml). Both SOD and MDA results clearly suggested that EK NPs effectively alleviated intracellular oxidative stress. Furthermore, we measured the changes of the ratio of reduced glutathione (GSH) to oxidized GSH (GSSG). As depicted in Figure 3E, H_2_O_2_ treatment caused a remarkable decrease in GSH/GSSG ratio to 7.70. This trend was successfully reversed by EK treatment and the GSH/GSSG ratio returned to baseline levels after treatment with 200 μg/ml EK (17.36 vs 18.9 in control group). Collectively, EK NPs exhibited effective intracellular ROS depletion and redox regulatory effects by reinstating the levels of redox-relevant enzymes and small molecules like GSH and GSSG.

### *In vitro* drug release and anti-inflammation of Cur-loaded EK NPs

**Figure 4.**
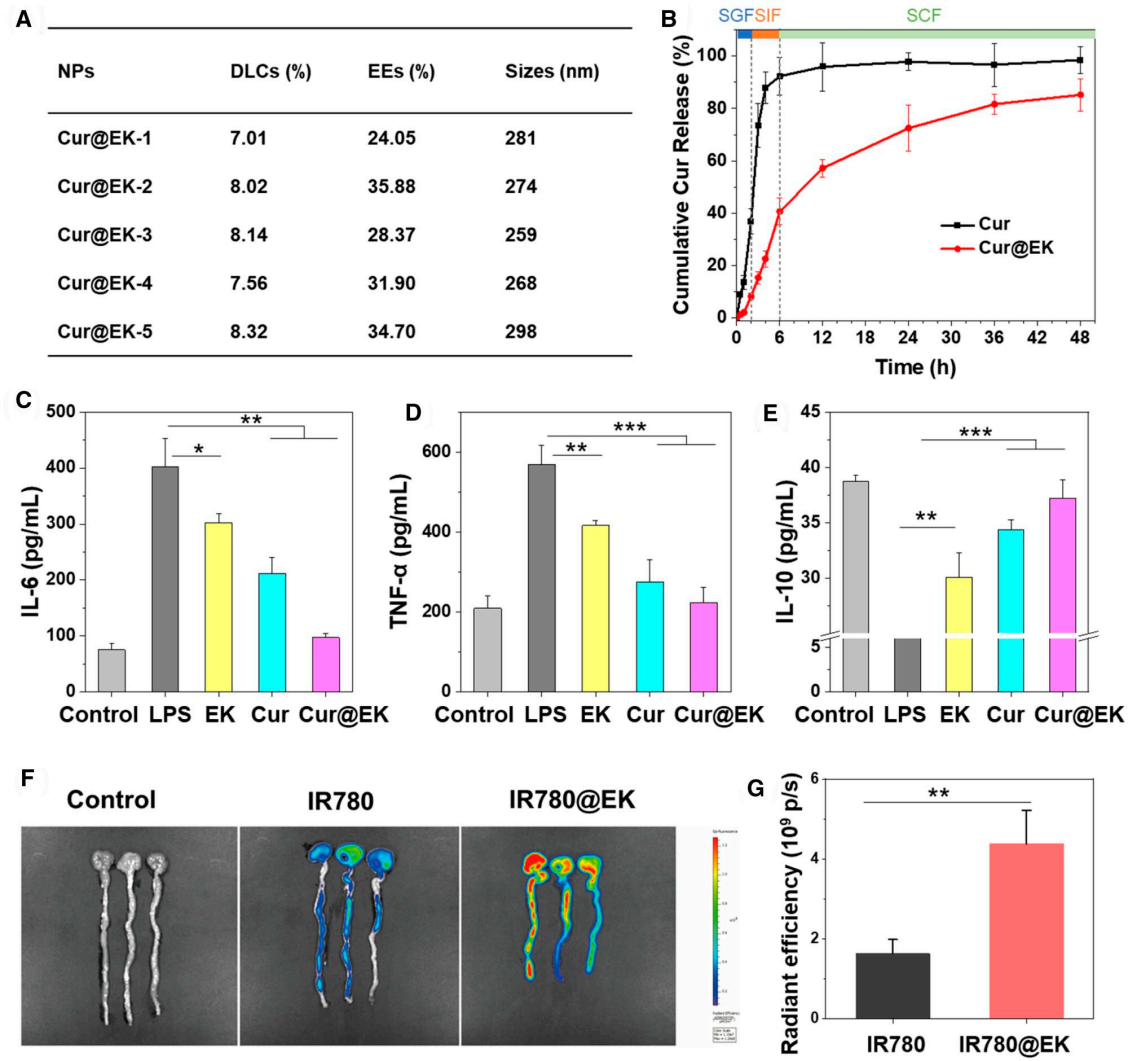
(**A**) Summary of Cur loading capacities and efficiencies by EK NPs. (**B**) *In vitro* drug release behavior of free Cur and Cur@EK NPs under gastrointestinal pH conditions (*n* = 3). (**C–E**) *In vitro* anti-inflammatory effect of Cur@EK in macrophages stimulated with LPS: IL-6 (C), TNF-α (D), and IL-10 (E) (*n *=* *5. * *P *<* *0.05, ** *P *<* *0.01, *** *P *<* *0.001). *Ex vivo* fluorescence images (**F**) and radiant efficiency (**G**) of colons from mice orally administrated with IR780 and IR780@EK (*n *=* *3. ** *P *<* *0.01).

Curcumin (Cur) is a multi-functional bioactive compound with anti-bacterial, anti-inflammation, and anti-cancer effects [10]. The anti-inflammatory effect of Cur is reported to inhibit IκBα kinase and Akt activation, thus preventing the activation of NF-κB [45, 46]. However, the poor solubility and low bioavailability of Cur had hampered its biomedical application. We utilized EK as a nanocarrier to load Cur for combinational anti-inflammation efficacy. Cur@EK NPs were prepared via a dialysis approach. The strong absorption of Cur@EK at 427 nm indicated successful encapsulation of Cur into EK NPs (Supplementary Figure S8). The Cur loading capacities in Cur@EK NPs varied from 7.01% to 8.32%, with encapsulation efficiencies ranging from 24.05% to 34.70% (Figure 4A). DLS results suggested that Cur-loaded EK NPs ranged from 259 to 298 nm (Figure 4A), larger than the corresponding blank nanoparticles. The increase in particle size of Cur@EK indicates that Cur participated in the self-assembly of EK nanoparticles. However, the size remains within a suitable range for both intravenous and oral administration. The zeta potentials of Cur-incorporated NPs were approximately −20.5 mV. Cur@EK-5 with a high Cur loading capacity was used for following investigations. SEM observation confirmed an increase in size after drug loading, and showed that Cur@EK retained a spherical morphology following Cur encapsulation (Supplementary Figure S9). The hydrodynamic size of Cur@EK were decreased with prolonged time in PBS and serum (Supplementary Figure S3), which was probably due to the sustained release of Cur.

The *in vitro* Cur release behavior of Cur@EK NPs was studied under simulated gastrointestinal pH conditions to explore the potential use as an oral nanomedicine (Figure 4B). Free Cur demonstrated rapid release at gastric and intestinal pHs, where approximately 92.2% was released at 6 h. In contrast, Cur@EK exhibited sustained release (40.1%) throughout gastric and intestinal pH conditions (0–6 h), suggesting that EK enabled prolonged controlled release of Cur. Under colonic pH condition (6–48 h), about 45.2% of Cur was released, implying that Cur@EK holds promise for improving the bioavailability of Cur in the colon and could be beneficial for treating colitis. Additionally, we evaluated the drug release profile of Cur@EK at pH 7.4 to simulate blood circulation conditions. As shown in Supplementary Figure S10, Cur exhibited a burst release, whereas Cur@EK demonstrated a sustained release, with approximately 82.5% of the drug released over 48 h.

To assess the potential anti-inflammatory properties of Cur@EK *in vitro*, macrophages were treated with LPS to induce inflammation through the TLR4 pathway, resulting in increased production of pro-inflammatory cytokines and ROS [47]. LPS stimulation notably raised IL-6 and TNF-α levels while reducing the anti-inflammatory cytokine IL-10 (Figure 4C–E). Macrophages treated with EK NPs showed reduced levels of IL-6 and TNF-α compared to LPS group. The anti-inflammatory effect was likely attributed to the anti-oxidant properties of EK NPs. Free Cur exhibited stronger *in vitro* anti-inflammatory effect than EK NPs. Notably, Cur@EK showed more robust anti-inflammatory effects *in vitro* than free Cur and EK, where the levels of IL-6, TNF-α, and IL-10 were similar to those of untreated macrophages. These results manifest that combining EK with Cur produces a synergistic anti-inflammatory effect *in vitro*.

### *In vivo* colitis alleviation assay

Before the *in vivo* anti-colitis study, we assessed the colon drug delivery efficiency by *ex vivo* fluorescence. A near infrared dye IR780 was used to label EK and the resulting IR780@EK was orally administrated using free IR780 as a control. At 24 h post oral administration, *ex vivo* colons were collected for fluorescence analysis using an IVIS Spectrum. As depicted in Figure 4F and G, weak colonic fluorescence was observed in free IR780 group while strong fluorescence in the IR780@EK group, indicating the enhanced colonic retention and bioavailability.

**Figure 5.**
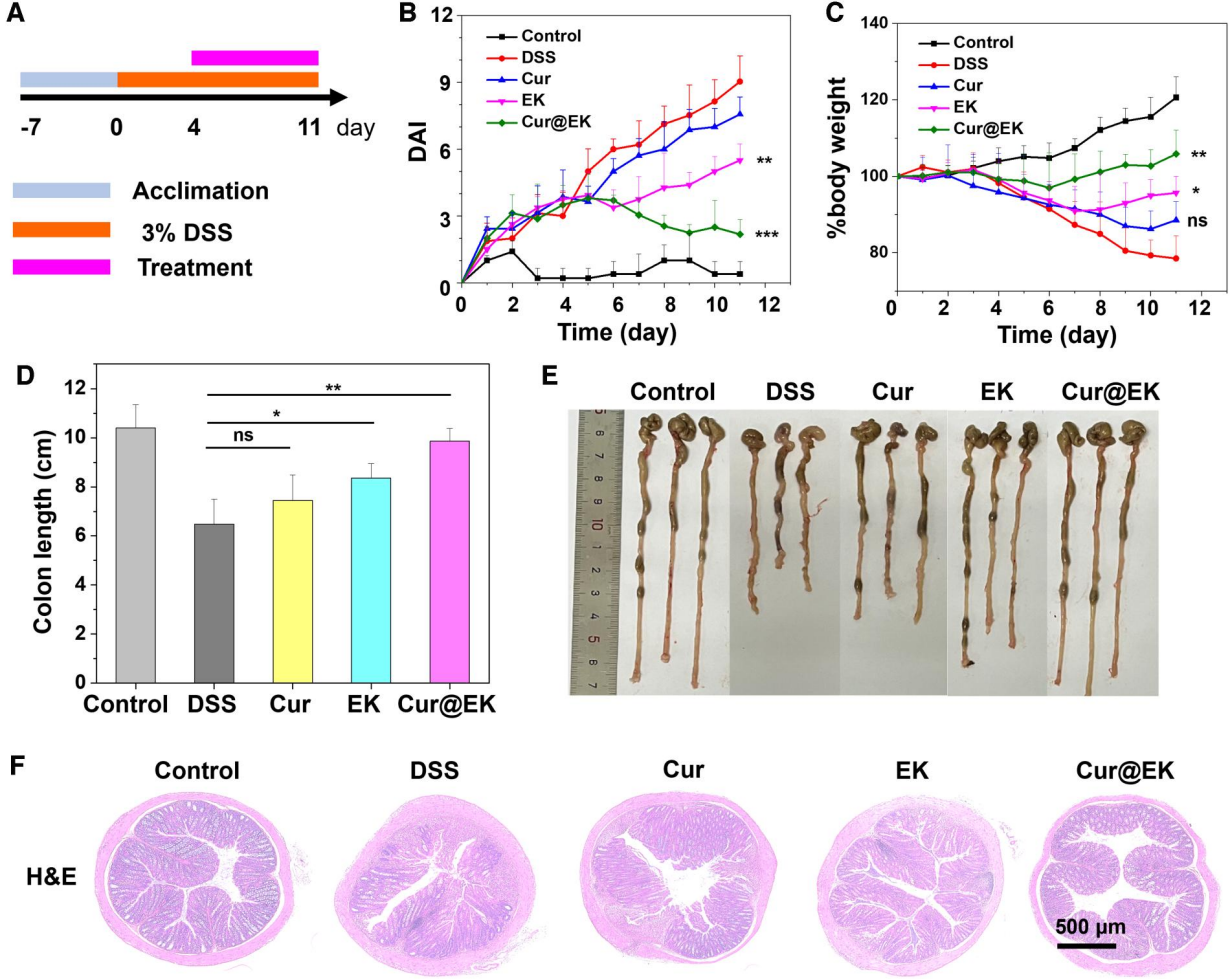
*In vivo* inflammation alleviation efficacy of Cur@EK in colitis mice induced by DSS. (**A**) Illustration of colitis model and therapy. Daily DAI scores (**B**), body weights (**C**), and colon lengths (**D**) (*n *=* *5. ns indicates not significant difference. * *P *<* *0.05, ** *P *<* *0.01, *** *P *<* *0.001). (**E**) Typical photographs of colons. (**F**) H&E-stained sections of colonic tissues.

Inspired by the anti-oxidant and anti-inflammatory effects and *in vivo* colonic retention of Cur@EK, we were interested in the *in vivo* effectiveness in reducing inflammation in a chemically induced colitis model. Balb/c mice were given DSS in their drinking water to establish ulcerative colitis model (Figure 5A). The colitis mice were gavaged with saline, curcumin, EK and Cur@EK. Healthy mice treated with DSS-free drinking water were used as a control. The disease activity index (DAI) and body weight of mice were recorded daily. Successful establishment of the colitis model was confirmed by severe weight loss and elevated DAI scores (Figure 5B and C). DSS group showed a DAI value of 9.0 and relative body weight of 78.52% on day 11. In contrast, the DAI values of Cur, EK and Cur@EK groups were 7.6, 5.5 and 2.2, respectively, suggesting the inflammation mitigation by treatments. Treatment with free Cur only obtained a relative body weight of 88.58% on day 11. In contrast, EK and Cur@EK treatments resulted in better weight restoration (Figure 5C), reaching a relative body weight of 95.68% and 105.87%, respectively. At the end point of therapy, colons were collected and the length of colons were measured to evaluate the therapeutic efficacy (Figure 5D and E). DSS insult caused significant shortening compared to control group (6.5 cm vs 10.4 cm). Cur showed unsatisfactory colon length shortening inhibition probably due to the low bioavailability. EK treatment exhibited longer colon length than Cur (8.4 cm vs 7.5 cm), suggesting the effective anti-oxidant therapy *in vivo*. Cur@EK treatment resulted in the least colon shortening and showed a colon length of 9.8 cm, indicating the synergistic *in vivo* anti-inflammatory effect of Cur and EK. Furthermore, H&E staining of colonic sections were employed to assess the therapeutic effect (Figure 5F). EK and Cur@EK treatments effectively alleviated the tissue damage caused by DSS.

**Figure 6.**
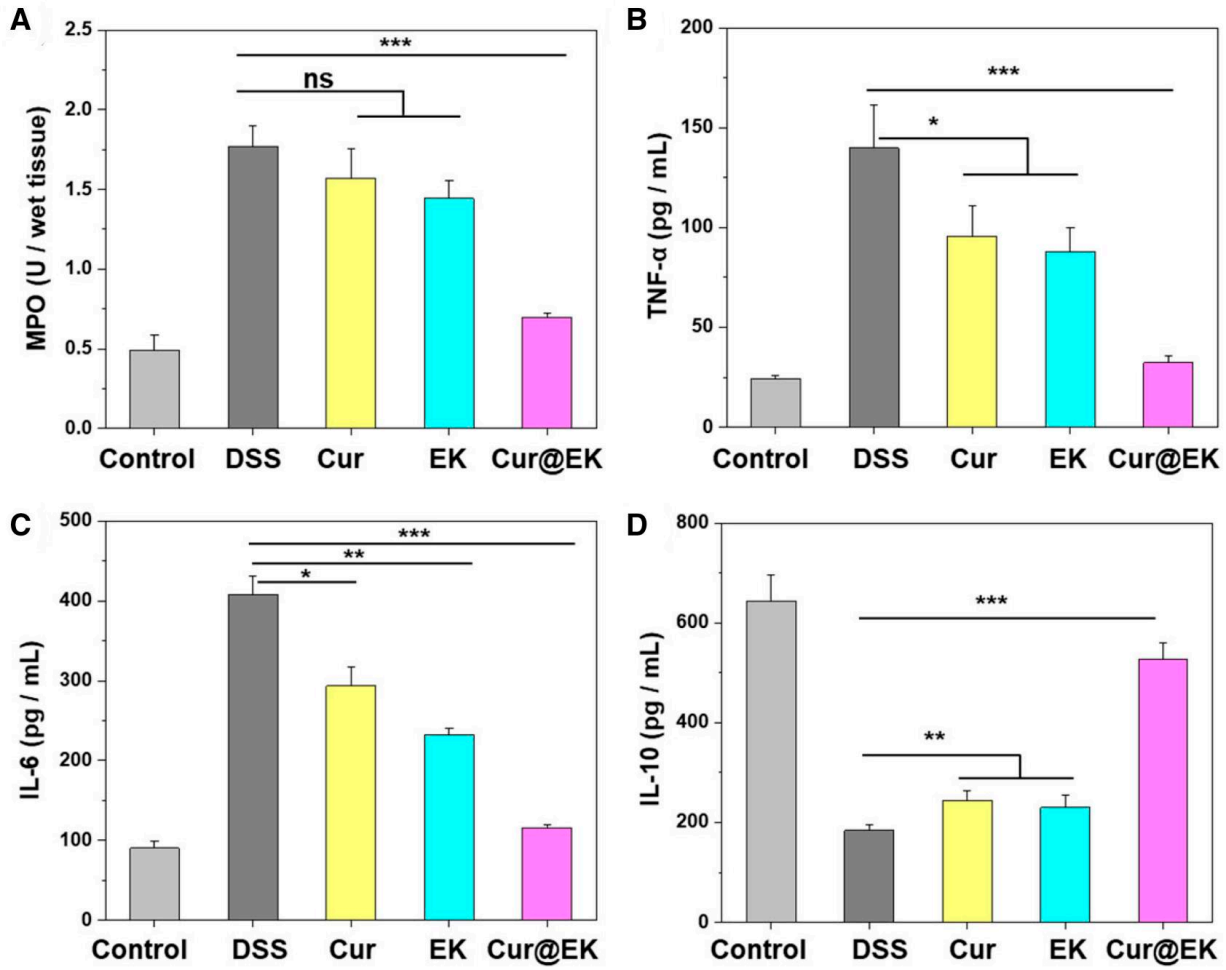
Evaluation of intestinal immune regulation in colitis mice. (**A**) MPO activity in the inflamed colons. Colonic levels of pro-inflammatory cytokines TNF-α (**B**) and IL-6 (**C**) as well as anti-inflammatory IL-10 (**D**). *n *=* *3. * *P *<* *0.05, ** *P *<* *0.01, and *** *P *<* *0.001.

Immune system dysregulation is a hallmark of chronic inflammation-associated diseases such as inflammatory bowel disease [48, 49]. Colonic MPO activity is a pivotal indicator of neutrophil infiltration and ROS level in ulcerative colitis [50]. For example, Nie *et al*. [51] ingeniously harnessed the MPO and/or ROS present in the inflamed colonic mucosa to trigger the *in situ* aggregation of phenol-rich nanoparticles, enabling targeted drug delivery to inflamed areas and enhancing therapeutic efficacy. As shown in Figure 6A, compared with DSS group, Cur@EK group showed a remarkable reduction in MPO activity, suggesting effective improvement in neutrophil infiltration and ROS upregulation. Furthermore, colonic level of inflammatory cytokines was measured using ELISA (Figure 6B–D). DSS administration led to a remarkable increase in pro-inflammatory cytokines IL-6 and TNF-α and decrease in anti-inflammatory cytokine IL-10, consistent with the *in vitro* results. Compared with DSS group, Cur and EK groups led to a significant decrease in IL-6 levels and an increase in IL-10. Importantly, Cur@EK showed lower levels of pro-inflammatory IL-6 and TNF-α, close to the level of healthy group, indicating that the combination of EK and Cur effectively improved the immune microenvironment in the inflamed colon [52].

**Figure 7.**
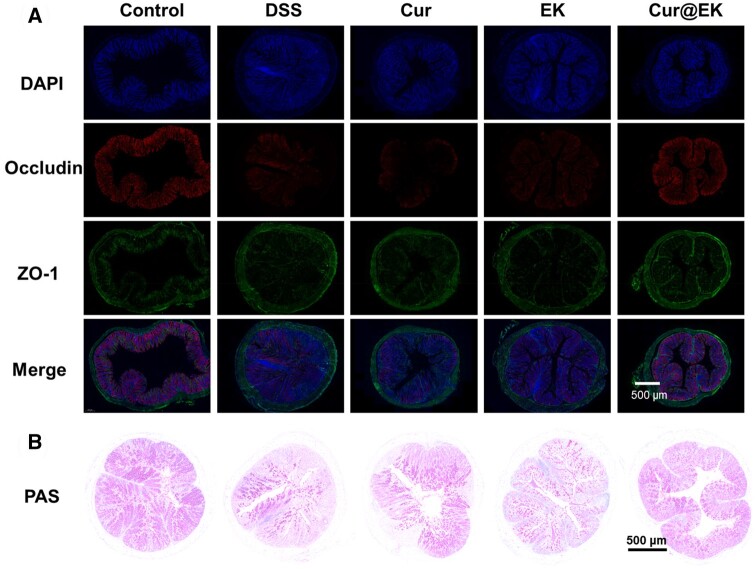
Epithelial repair evaluation in colitis mice. (**A**) Levels of colonic Occludin and ZO-1 analyzed by immunofluorescence staining. (**B**) PAS staining images of inflamed colon sections.

To further assess the epithelial repair effect of Cur@EK, the expression level of tight junctions Occludin and ZO-1 was evaluated by immunofluorescence staining. As shown in Figure 7A, DSS led to a significant decrease of Occludin and ZO-1 expression in epithelial tissue. In stark contrast, Cur@EK treatment exhibited notably increased levels of tight junction proteins, suggesting the significant role in repairing epithelial damage that is widely observed in ulcerative colitis. The periodic acid-Schiff (PAS) staining was used to visualize and evaluate the presence and distribution of mucus in the colon. Figure 7B depicted that Cur@EK treatment induced efficient mucus secretion by goblet cells, indicating the reconstruction of mucus barrier and restoration of goblet cell amounts. Additionally, H&E staining of major organs indicated that no obvious organ toxicity was observed (Supplementary Figure S11), indicating the satisfactory biocompatibility of EK and Cur@EK. Taken together, Cur@EK as an oral nanomedicine exhibited a combinational anti-inflammatory effect in colitis by increasing the bioavailability of Cur and leveraging the antioxidant and anti-inflammatory properties of both EK and Cur.

### AKI therapy assay

**Figure 8.**
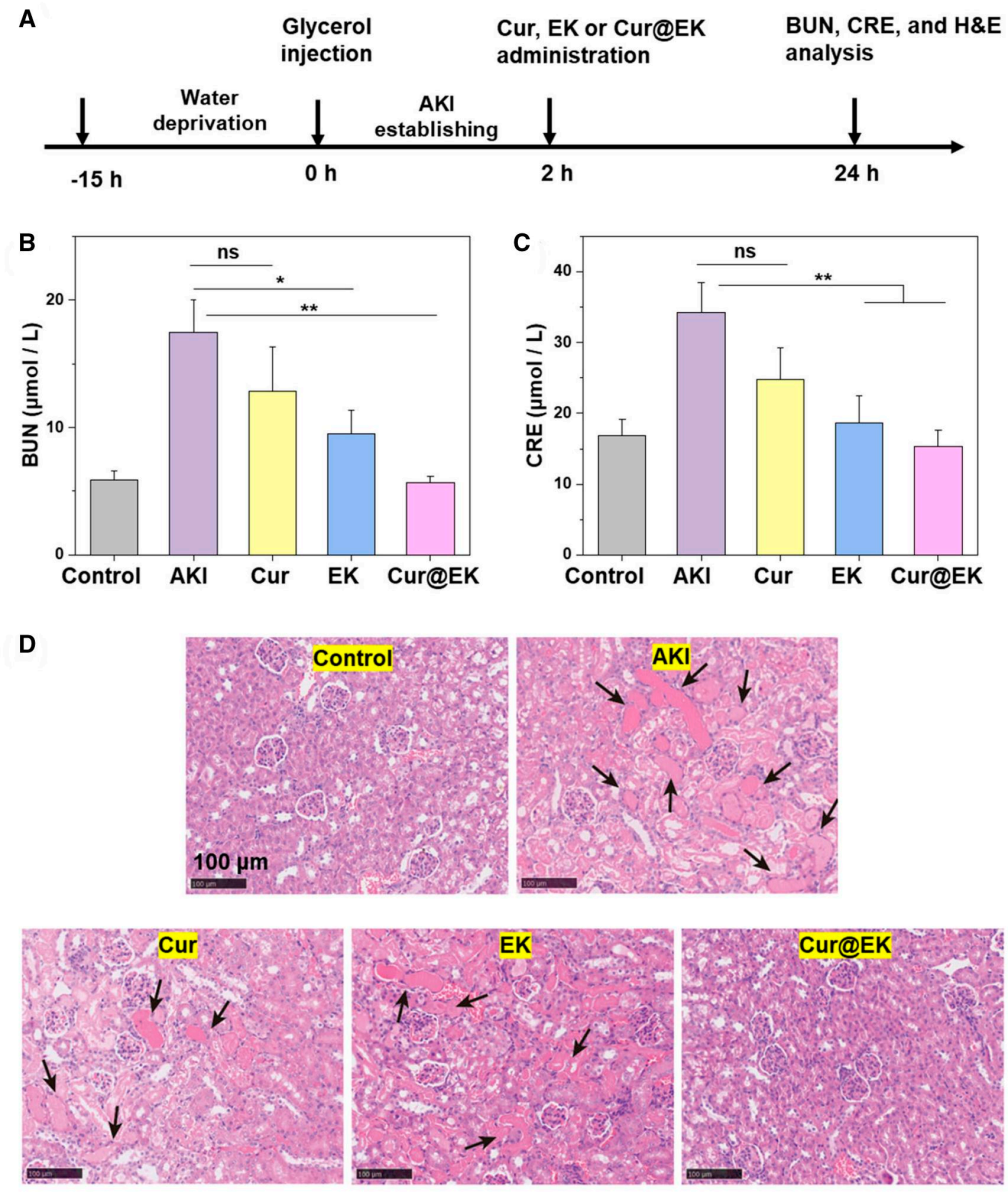
Therapeutic effect of Cur@EK in acute kidney injury. (**A**) The experimental protocol of AKI model establishment and treatment. Serum BUN (**B**) and CRE (**C**) levels in different groups (*n *=* *3. * *P *<* *0.05 ** *P *<* *0.01). (**D**) H&E staining images of kidneys; the black arrows indicate tubular damages.

The therapeutic effect of Cur@EK in AKI was studied. Intramuscular injection of glycerol was used to establish a mouse model with AKI (Figure 8A) [33]. Saline (AKI group), Cur, EK and Cur@EK were intravenously injected at 2 h post glycerol administration. In the context of AKI, blood urea nitrogen (BUN) and serum creatinine (CRE) are critical markers for assessing kidney function. Kidney is responsible for filtering BUN and CRE, and the elevated levels of both indicate a decline in kidney function, which is characteristic of AKI [53]. The serum levels of BUN and CRE after treatments were shown in Figure 8B and C. Glycerol resulted in a notable increase in BUN and CRE levels, indicating acute kidney injury. However, EK and Cur@EK treatments led to a significant reduction in BUN and CRE levels compared to the AKI group, indicating efficient mitigation of kidney injury. Noteworthily, Cur@EK treatment nearly normalized the levels of serum BUN (5.65 μmol/L) and CRE (15.3 μmol/L), demonstrating the most potent anti-inflammatory effect in AKI. Furthermore, H&E staining was carried out to evaluate the therapeutic effect of Cur@EK (Figure 8D). As expected, there were many damaged renal tubules in the AKI group. In stark contrast, Cur@EK treatment effectively alleviated kidney injury and minimal tubule damage was observed, which was in agreement with the results of serum kidney injury indicators. Overall, these results clearly manifested that intravenous administration of nanoscale Cur@EK could be used to treat AKI.

**Figure 9.**
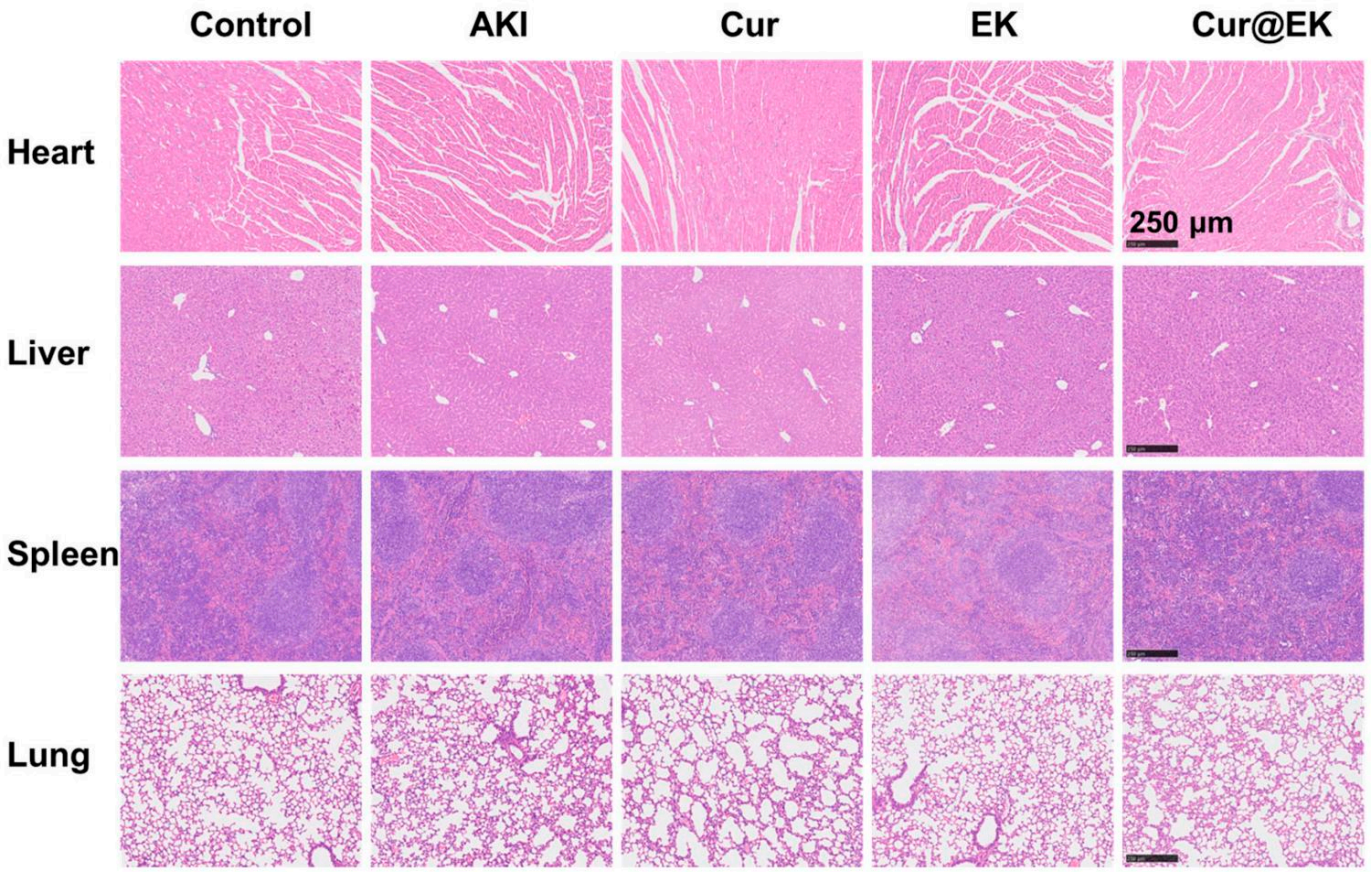
H&E-stained heart, liver, spleen, and lung sections of AKI mice.

Lastly, the biosafety properties of EK and Cur@EK were studied by assessing their toxicity in major organs. As shown in Figure 9, no notable tissue damage was observed in the major organs (heart, liver, spleen and lung) in the EK and Cur@EK groups, highlighting the exceptional biocompatibility of these nanoformulations. These findings clearly demonstrated that Cur@EK is a safe and effective nanoformulation to manage AKI.

## Conclusion

In this study, we developed an EGCG-based nanoparticle EK to improve the oral and systemic bioavailability and anti-inflammatory effect of curcumin in ulcerative colitis and acute kidney injury. EK nanoparticles showed significant radical scavenging capability, intracellular ROS reduction, and regulation of the redox microenvironment. Importantly, EK served not only as a carrier for Cur but also enhanced the *in vitro* anti-inflammatory effect *in vitro*. In the *in vivo* studies, Cur-loaded EK nanoparticles exhibited efficient colon retention and effective mitigation of acute colitis after oral administration. Besides, intravenously injected Cur@EK showed enhanced inflammation mitigation compared with free EK in a murine model of acute kidney injury. Moreover, this nanomedicine exhibited excellent biocompatibility both *in vitro* and *in vivo*. In summary, Cur@EK represents a promising anti-inflammatory nanosystem worthy of further exploration in diverse inflammation-associated disorders.

## Supplementary Material

rbae122_Supplementary_Data
