# Supplementary material for: Curcumin-incorporated EGCG-based nano-antioxidants alleviate colon and kidney inflammation via antioxidant and anti-inflammatory therapy
Source: Regen Biomater. 2024 Oct 17;11:rbae122. doi: 10.1093/rb/rbae122 (PMC11558062; doi:10.1093/rb/rbae122)
Supplement: rbae122_Supplementary_Data [file rbae122_supplementary_data.docx]

Supporting information

**Curcumin-incorporated EGCG-based nano-antioxidants alleviate colon and kidney inflammation via antioxidant and anti-inflammatory therapy**

Qingqing Pan,^a^ Li Xie,^a^ Huang Zhu,^a^^*^ Zhihui Zong,^b*^ Di Wu,^c^ Rong Liu, ^a^ Bin He,^d^ Yuji Pu^d*^

^a^ School of Preclinical Medicine, Chengdu University, Chengdu 610106, China

^b^ Department of Pharmaceutical Engineering, Bengbu Medical University, Bengbu 233030, China

^c^ Meat Processing Key Laboratory of Sichuan Province, School of Food and Biological Engineering, Chengdu University, Chengdu 610106, China

^d^ National Engineering Research Center for Biomaterials, College of Biomedical Engineering, Med-X Center for Materials, Sichuan University, Chengdu 610064, China

**Figure S1.** Zeta potentials of EK nanoparticles prepared at different feeding EGCG/Lys molar ratios.


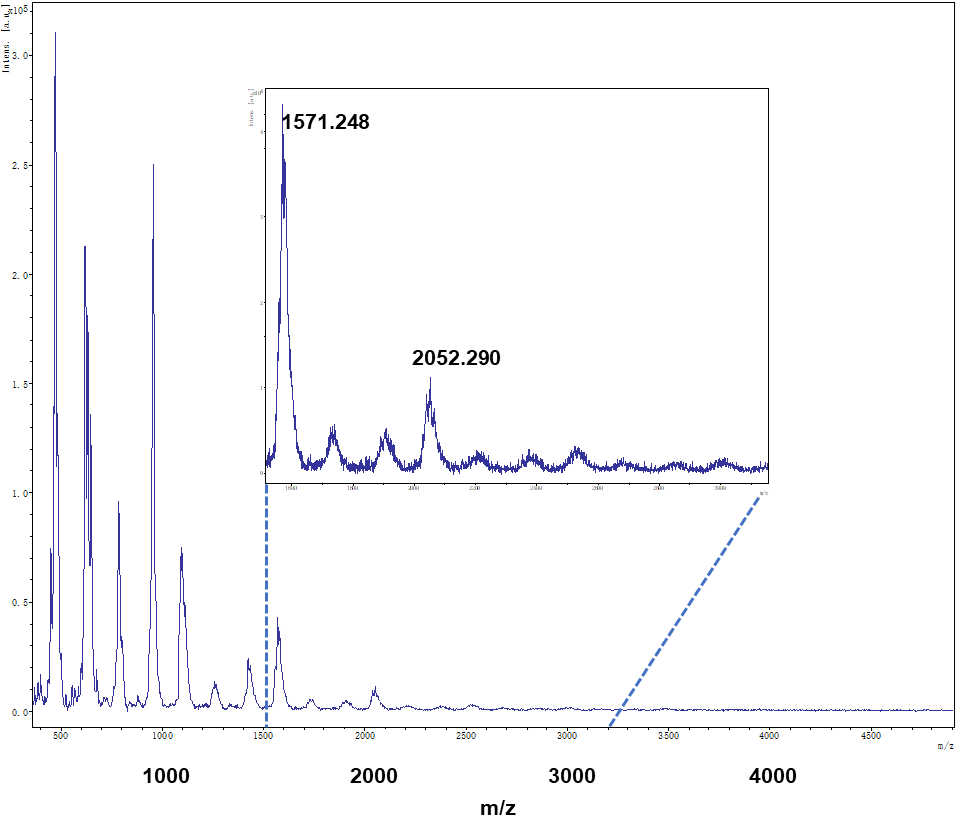


**Figure S2.** MALDI-TOF MS spectrum of EK-5 NPs.


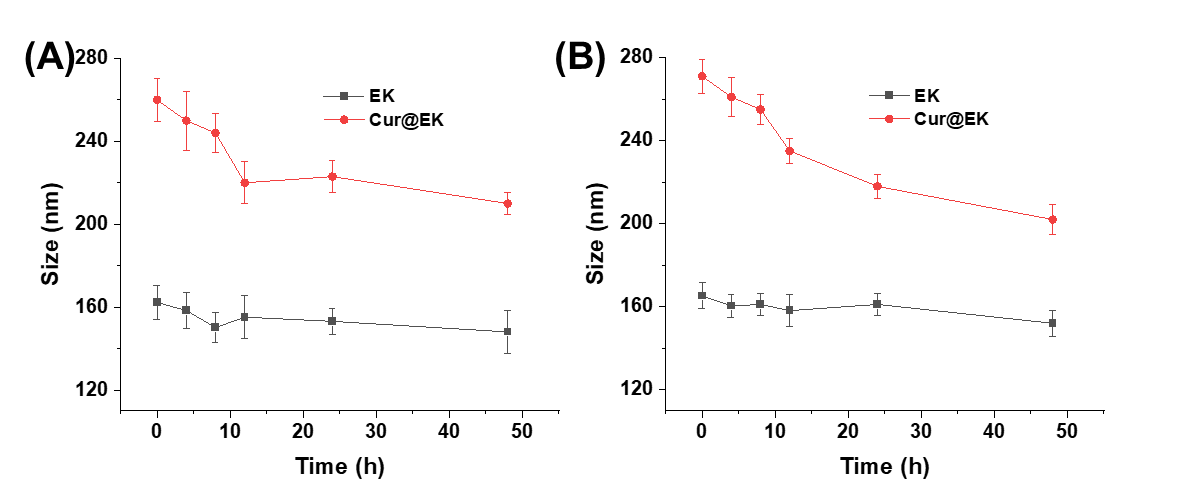


**Figure S3.** The size change of EK and Cur@EK in PBS without (A) and with (B) 10% fetal bovine serum.

**Figure S4.** Viabilities of L929 cells upon a 24-h treatment of EK NPs.

**Figure S5.** Viabilities of RAW264.7 cells after treated with EK NPs for 24 h.


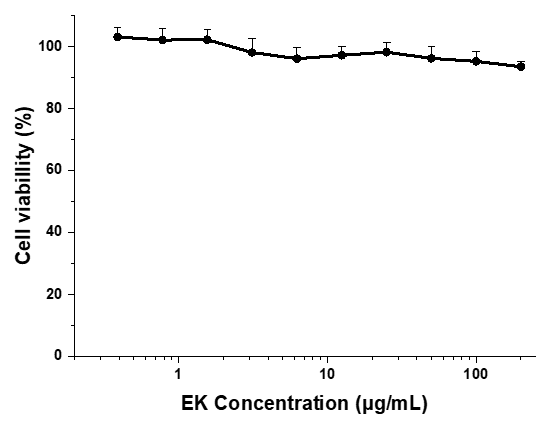


**Figure S6.** Viabilities of NCM460 cells after co-incubation with EK-5 nanoparticles for 24 h.


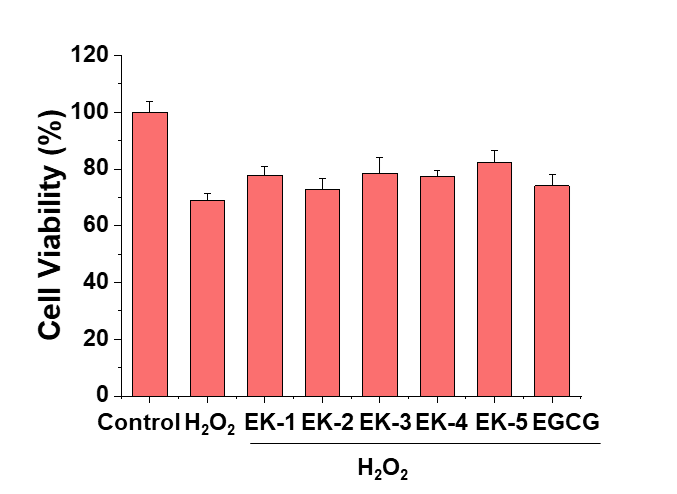


**Figure S7.** Viabilities of RAW264.7 cells after co-incubation with H_2_O_2_ and EK NPs.


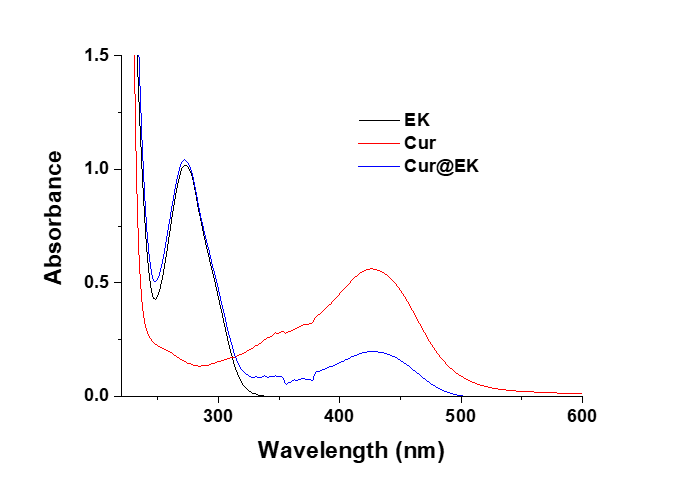


**Figure S8.** UV-vis absorption of Cur@EK.


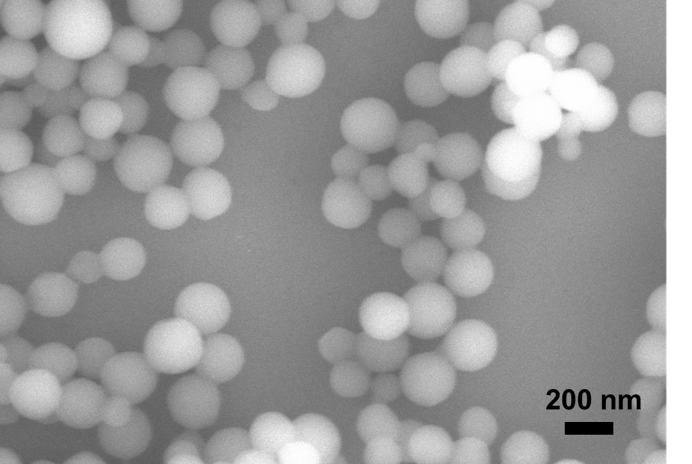


**Figure S9.** SEM-image of Cur@EK.

**Figure S10.** In vitro drug release of Cur@EK at pH 7.4.


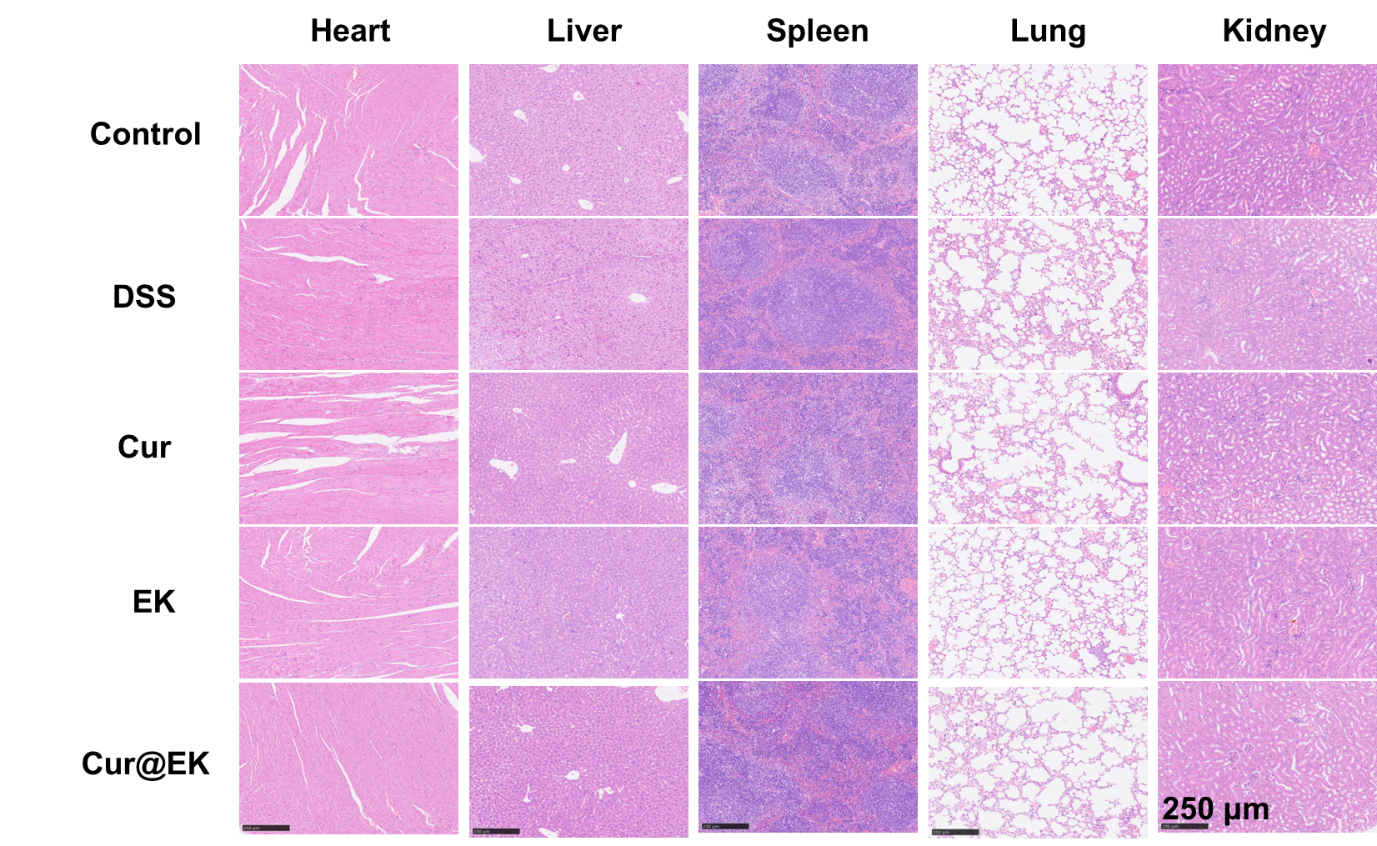


**Figure S11.** H&E staining sections of major organs from colitis mice.

**Table S1. Preparaing parameters for the synthesis of EK nanoparticles.**

| **NPs** | **EGCG (mg)/water (mL)** | **HCHO (μL)** | **Lys (mL, 14.6 mg/mL, pH 10.0)** | **pHs at the end point** |
| --- | --- | --- | --- | --- |
| **EK-1** | 137.5 mg/19 mL | 60 | 1 mL | 5.43 |
| **EK-2** | 137.5 mg/18.5 mL | 60 | 1.5 mL | 5.69 |
| **EK-3** | 137.5 mg/17 mL | 60 | 3 mL | 7.06 |
| **EK-4** | 137.5 mg/14 mL | 60 | 6 mL | 8.02 |
| **EK-5** | 137.5 mg/11 mL | 60 | 9 mL | 8.65 |
